# Supplementary material for: Cortical Microstructural Variations Correlate With Individual Differences in Gamified Exploration–Exploitation Behaviours
Source: Comput Psychiatr. 2026 Aug 5;10(1):121–41. doi: 10.5334/cpsy.164 (PMC13450271; doi:10.5334/cpsy.164)
Supplement: Supplementary file 1 [file cpsy-10-1-164-s1.pdf]

Supplementary Information

Supplementary Figure 1

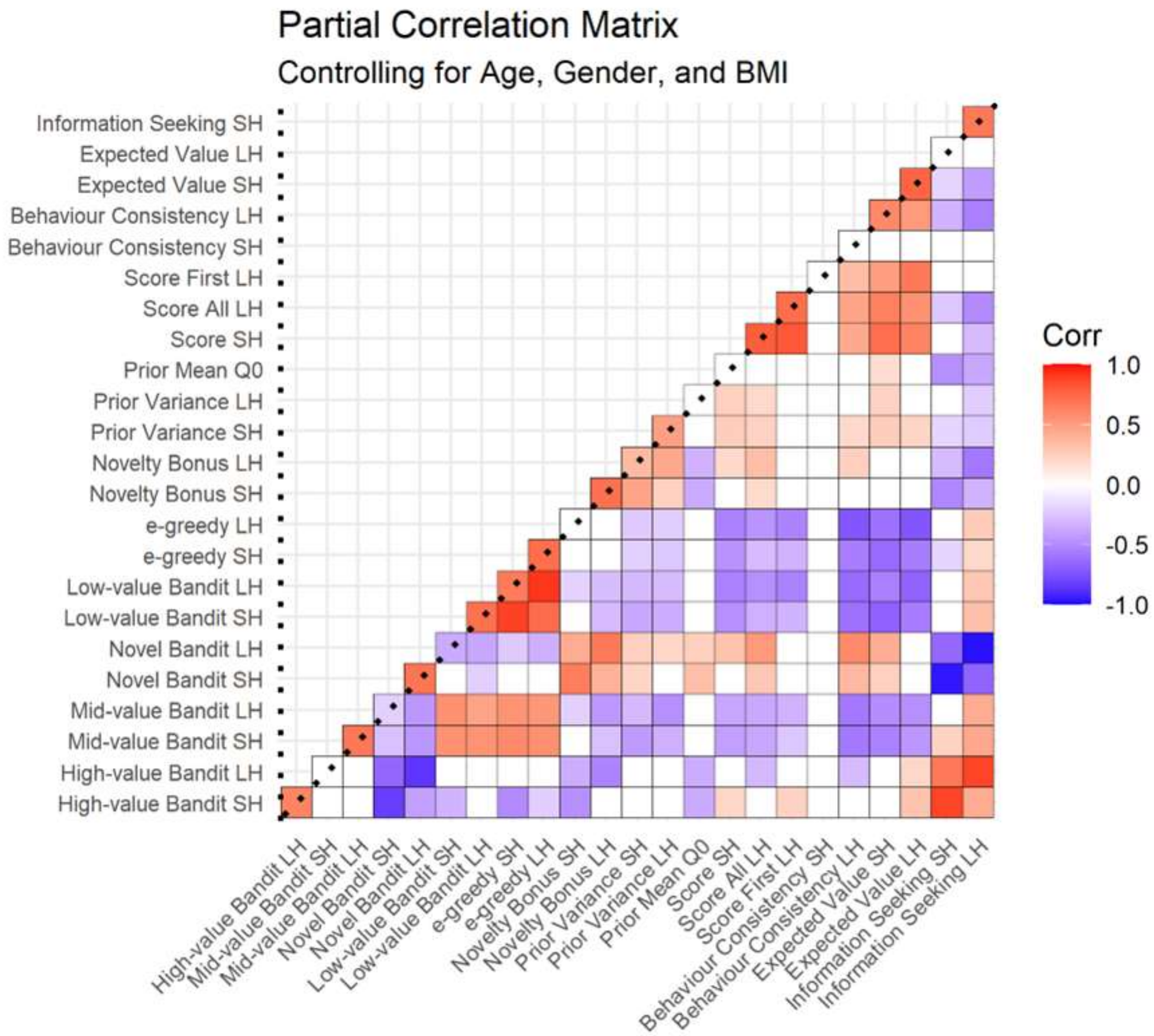

SF1 Partial correlation matrix detailing Spearman's correlations between choice behaviour, model heuristics, and Thompson model parameter estimates, corrected for age, gender, and body mass index (BMI).

Supplementary Information 1: Associations between behavioural metrics and transdiagnostic psychiatric factors

To further interrogate the origins of participants' exploration-exploitation behaviours, we examined the association between participants' choice behaviours and model heuristics, and multi-level mental health factors. Factor scores for each participant denoting mental health constructs were drawn from item-level exploratory factor analyses (EFA) performed on responses from eight mental health self-report surveys from the full Visceral Mind Project cohort of 566 participants. Survey instruments spanned autism spectrum (AQ10), ADHD (ASRS), depression (MDI and PHQ9), somatic symptoms (PHQ15), stress (PSS), social anxiety (SIAS), and trait anxiety (STAI trait) (Banellis et al., 2025). This multi-level factor solution revealed 11 lower-level factors and two higher-level factors. The higher-level factor 'ADHD & Somatic' encompassed four lower-level factors: negative thoughts, restlessness, somatic symptoms, and impulsivity. The second higher-level factor 'affective symptoms' encompassed six lower-level factors: social anxiety, self-confidence, sleep, depression, stress, and autism (**Supplementary Figure 2**). The eleventh lower-level factor was inattentiveness, which we did not examine in this study (see Banellis et al. 2025 for further information regarding EFA methods).

We then applied Spearman's rank partial correlations comparing behavioural metrics to mental health factors, correcting for age, gender, and body mass index (BMI), and correcting for multiple comparisons (FDR correction method, Benjamini-Hochberg procedure at < .05). We found a significant positive correlation between the affective symptoms higher-level factor and participants' prior mean Q_0, i.e., the prior beliefs held by a participant about a bandit's mean value before seeing any samples (**Supplementary Figure 3**). This suggests that participants who exhibited greater affective symptoms of anxiety and depression were more likely to hold beliefs that unexplored bandits would yield higher rewards. This finding differs from previous work by Dubois and Hauser (2022) who instead found significant positive associations between the anxious-depression factor and participants' novelty bonuses (Dubois and Hauser 2022).

We then conducted post-hoc Spearman's rank partial correlations to further examine the relationship between the prior mean Q_0 and the six lower-level factors encompassed within the affective symptoms higher-level factor, again correcting for age, gender, and BMI, and correcting for multiple comparisons (FDR correction). This revealed a significant positive correlation between the prior mean Q_0 and the social anxiety lower-level factor, in addition to a significant negative correlation between the prior mean and the self confidence factor (negative

loading) (**Supplementary Figure 3**). Together, these findings indicate that participants who experience greater social anxiety and reduced confidence in oneself may place inflated value expectations upon unknown options.

Supplementary Information 2: Detailed Task Instructions

Prior to starting the task, participants underwent a short task tutorial with the following instructions, alongside visual examples of bandits (trees bearing apples) and rewards (apples):

"*Apples come in different shades and sizes. You need to help us collect the BIGGEST ones before sunset. You can only pick apples until sunset. Sometimes you will start at noon and will pick six apples, sometimes you will start in the afternoon and will pick only one apple. On each day you will collect apples from new trees. Some of the trees are better than others. To help you, some apples were already picked up before you arrived. These are the three different types of trees. Each tree produces an apple with a corresponding colour, but it's their size that matters! For example, if we look at the apples produced, the yellow tree seems to be better on this day because it produces bigger apples ON AVERAGE. This is a big apple from the yellow tree. This is a medium apple from the red tree. This is a small apple from the yellow tree. This is a small apple from the blue tree. And finally this is a big apple from the blue tree. You will press the key 1, 2, or 3 to select the tree you wish to pick an apple from. If the sun is here, it is still early and you will be able to pick six apples. If the sun is almost set, you will only be able to pick one apple. At the end of each day, you will see the amount of juice extracted from the apples you collected. The juice will be poured in a small glass if one apple was picked, and in a big glass if six apples were picked.*"

Supplementary Figure 2

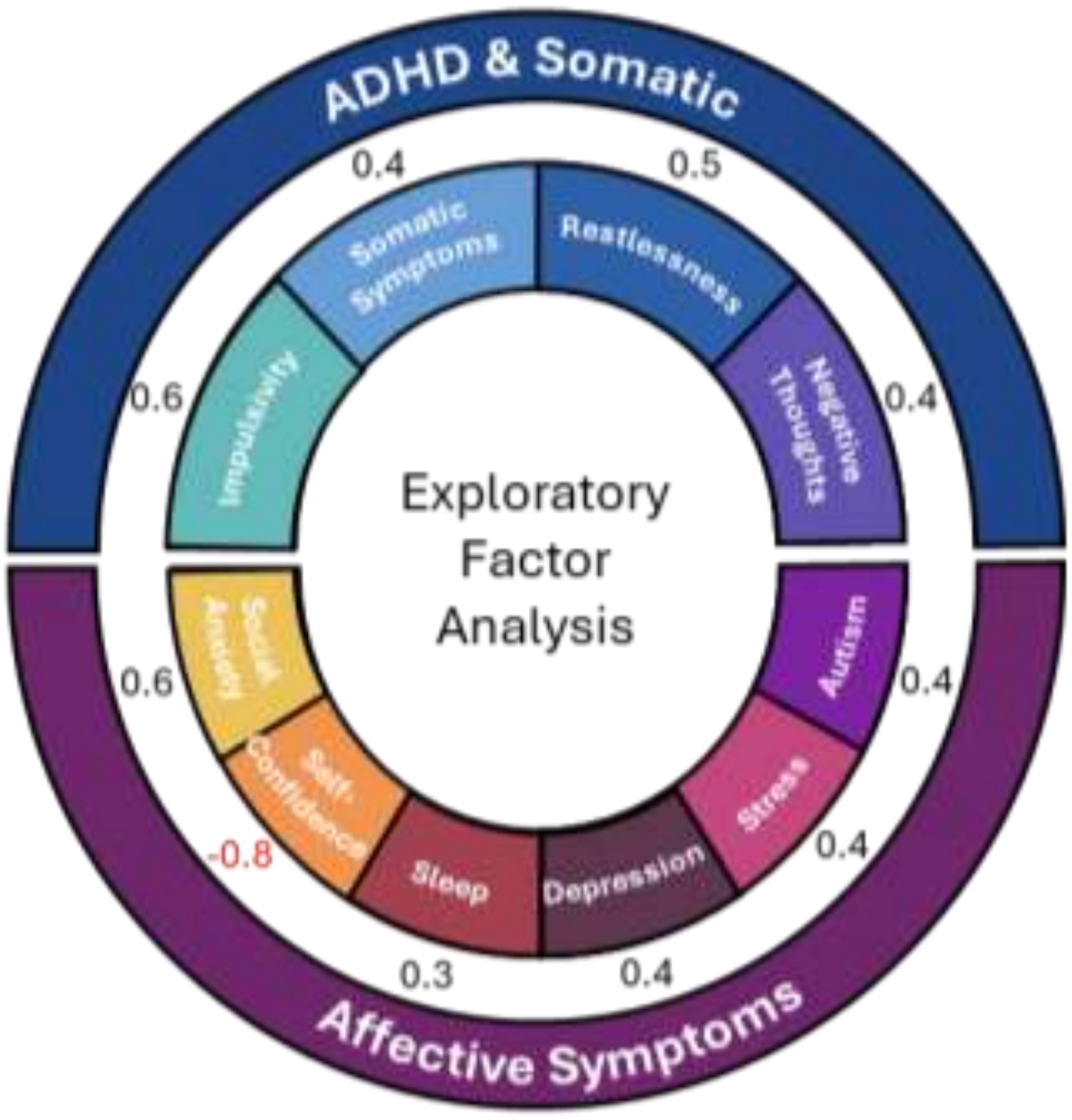

SF2 Multi-level factor solution derived from the responses in eight mental health surveys of 566 participants in the Visceral Mind Project (Banellis et al. 2025). Outer circle denotes the two higher-level factors: affective symptoms and ADHD & somatic. Inner circle denotes the lower-level factors: social anxiety, self-confidence, sleep, depression, stress, autism, negative thoughts, restlessness, somatic symptoms, and impulsivity. The eleventh lower-level factor, inattentiveness, was not encompassed within either of the two higher-level factors and was not examined here. Numbers denote factor loadings for each lower-level factor. For more details regarding the exploratory factor analysis (EFA) methods, see Banellis et al. (2025).

Supplementary Figure 3

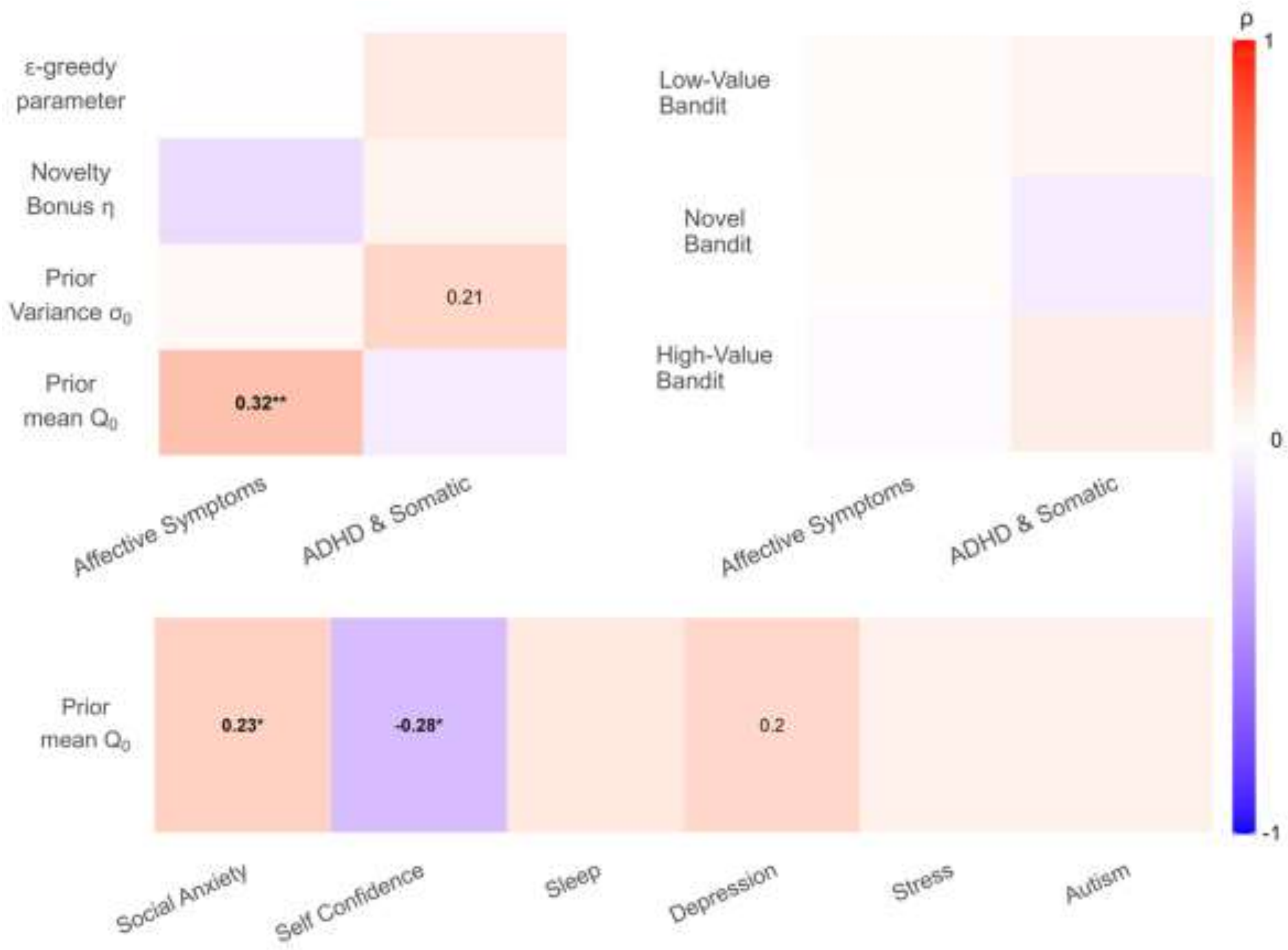

SF3 *Left:* The exploratory factor analysis (EFA)-derived 'Affective Symptoms' higher-order factor was significantly correlated with prior mean Q_0, i.e., participants' prior beliefs about a bandit's mean value before seeing any samples (r(107) = 0.32, $p_{unc} < .001$, $p_{cor} = .00667$; FDR-corrected partial correlations correcting for age, gender, and BMI). *Right:* There were no significant correlations between either of the EFA higher-order factors and participants' behaviour (i.e., the frequency of picking the low-value, novel, or high-value bandits). *Bottom:* FDR-corrected post-hoc partial correlations correcting for age, gender, and BMI revealed that the association between the affective symptoms higher-order factor and prior mean Q_0 is driven by significant positive correlation with the social anxiety factor (r(107) = 0.23, $p_{unc} = .0105$, $p_{cor} = .0315$) and significant negative correlation with the self confidence factor (r(107) = -0.28, $p_{unc} = .00248$, $p_{cor} = .0149$). For significant uncorrected correlations, *p* values are printed on the heatmaps, and *p* values for significant corrected correlations are indicated in bold with asterisks (*$p < .05$, **$p < .01$, ***$p < .001$).

Supplementary Figure 4

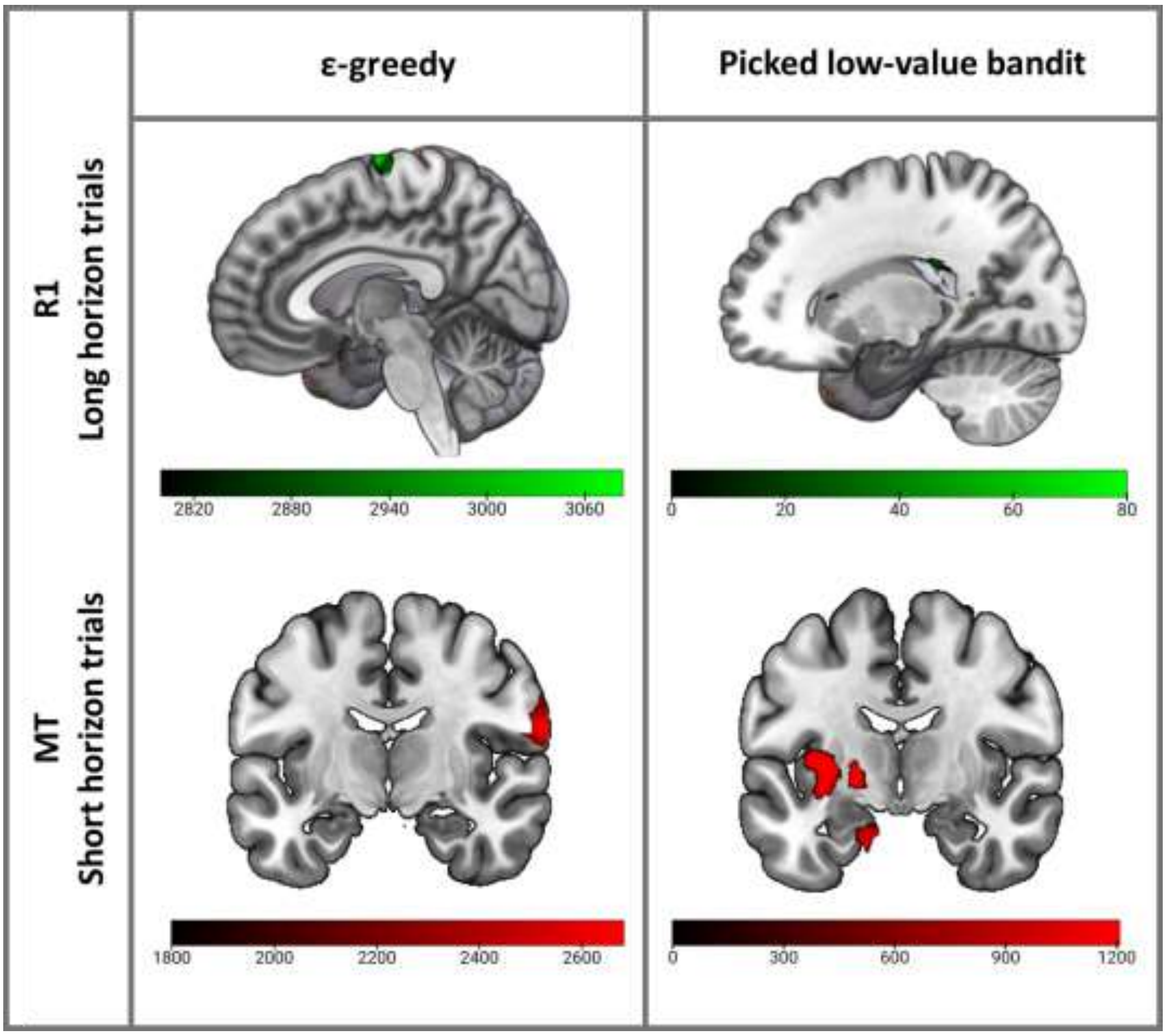

SF4 Comparison of main VBQ findings examining associations between cortical microstructures and ε-greedy parameter estimates (left), versus the simpler behavioural metric of bandit selection frequency (right).

Supplementary Figure 5

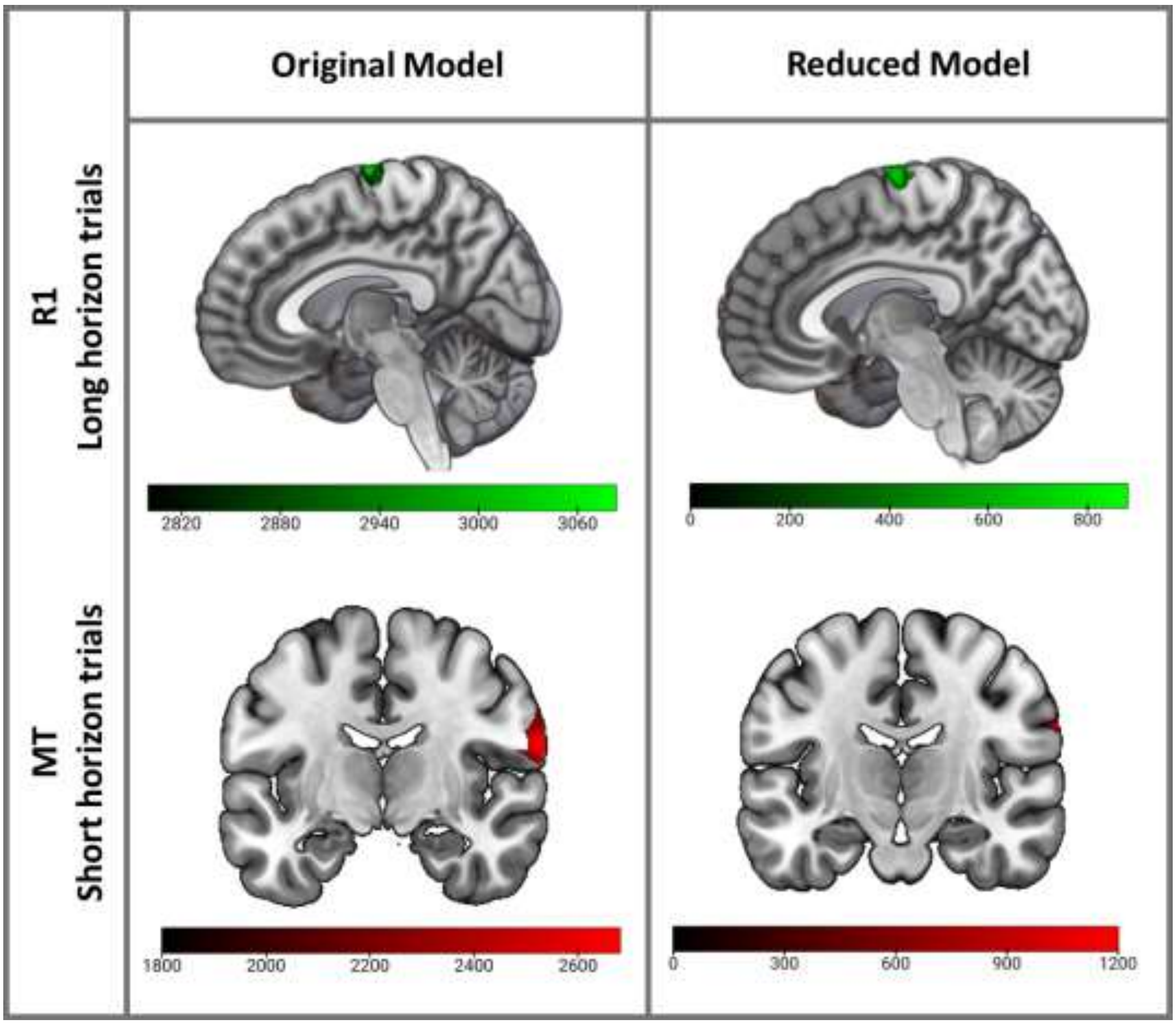

SF5 Comparison of main VBQ findings for the original model comprising 16 regressors of interest and four nuisance covariates (age, gender, BMI, and TIV), and a reduced model comprising ε-greedy for short and long horizon trials only, plus the same four nuisance covariates.

Supplementary Figure 6

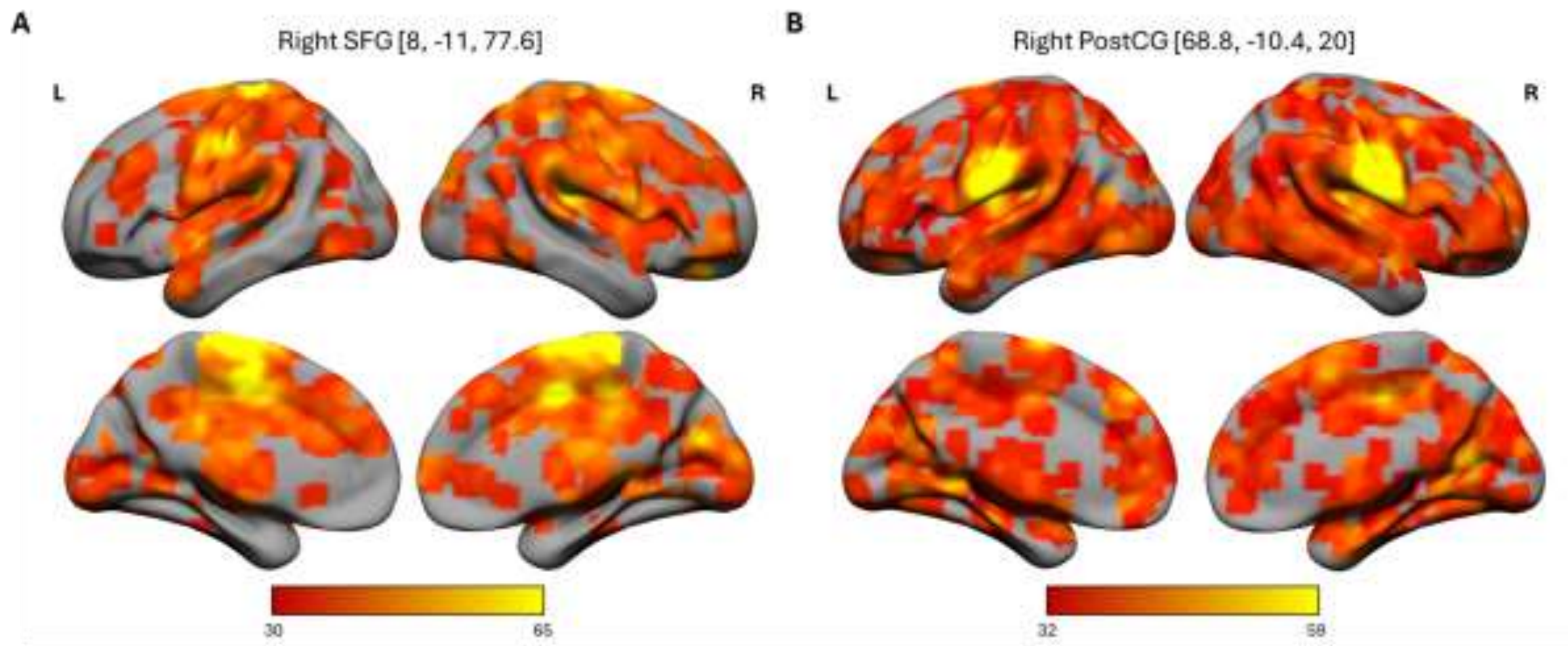

SF6 Meta-analytic analyses for right SFG and right PostCG seed regions identified through VBQ analyses, displaying areas of significant coactivation. SFG = superior frontal gyrus; PostCG = postcentral gyrus.

Supplementary Tables

Supplementary Table 1

R1 Whole Brain Clusters for Raw Behavioural Data							
**Region**	**k**	**p(FWE-corr)**	**p(uncorr.)**	**TFCE**	**x**	**y**	**z**
Left lateral ventricle	1	1.00	0.049	132.53	-24.0	-52.8	13.6
Left lateral ventricle	1	1.00	0.049	132.04	-23.2	-51.2	12.8
Right lateral ventricle	19	1.00	0.043	123.99	34.4	-42.4	3.2
Right lateral ventricle	82	1.00	0.030	81.92	23.2	-30.4	24

Supplementary Table 1: Summary of whole brain VBQ results: positive correlation between R1 map values and low-value bandit selection frequency in long horizon trials. Clusters are ordered by FWE-corrected p value.

Supplementary Table 2

MT Whole Brain Clusters for Raw Behavioural Data							
**Region**	**k**	**p(FWE-corr)**	**p(uncorr.)**	**TFCE**	**x**	**y**	**z**
Left putamen	3949	0.200	0.015	1207.01	-33.6	-6.4	2.4
Left pallidum	657	0.208	0.018	1177.95	-18.4	-10.4	-2.4
Right basal forebrain	3433	0.211	0.016	1167.07	20.8	2.4	-15.2
Right frontal operculum	1771	0.241	0.011	1073.67	40.8	16.8	10.4
Right posterior orbital gyrus	465	0.246	0.020	1057.30	24.0	16.0	-14.4
Left parahippocampal gyrus	3517	0.268	0.013	997.49	-12.8	-7.2	-29.6
Left ventral DC	549	0.295	0.019	932.45	-12.8	-20.0	-18.4
Left parahippocampal gyrus	1	0.313	0.019	890.73	-20.0	30.4	-28.8
Left parahippocampal gyrus	10	0.316	0.019	884.47	-24.0	-26.4	-29.6
Right OrIFG	1149	0.335	0.017	846.34	48.0	29.6	-10.4

Supplementary Table 2: Summary of top ten whole brain VBQ results: negative correlation between MT Saturation and low-value bandit selection frequency in short horizon trials. Clusters are ordered by FWE-corrected p value. DC = diencephalon; OrIFG = orbital part of inferior frontal gyrus.

Supplementary Table 3

Name	z-score	Posterior prob.	Func. conn. (r)	Meta-analytic coact. (r)
foot	8.35	0.95	0.48	0.19
muscle	6.02	0.92	0.23	0.13
suppressed	5.95	0.92	0.01	0
substantia	5.13	0.92	-0.05	-0.01
motor premotor	4.82	0.91	0.28	0.12
distractor	4.75	0.91	-0.01	-0.01
electrical	5.12	0.9	0.19	0.16
cortex dorsal	4.58	0.9	0.02	0.01
midbrain	4.9	0.88	-0.06	0
coordination	4.55	0.88	0.27	0.22

Supplementary Table 3: Top ten meta-analysis map associations for the right SFG, ordered by posterior probability.

Supplementary Table 4

Name	z-score	Posterior prob.	Func. conn. (r)	Meta-analytic coact. (r)
postcentral gyrus	6.54	0.89	0.14	0.2
pitch	5.57	0.89	0.19	0.26
speech production	5.4	0.89	0.35	0.44
taste	5.04	0.89	0.02	0.09
vocal	4.78	0.89	0.24	0.31
postcentral	5.49	0.85	0.1	0.26
multisensory	4.38	0.85	0.07	0.13

Supplementary Table 4: Meta-analysis map associations for the right postcentral gyrus, ordered by posterior probability.
