# Supplementary material for: Cortical Microstructural Variations Correlate With Individual Differences in Gamified Exploration–Exploitation Behaviours
Source: Comput Psychiatr. 2026 Aug 5;10(1):121–41. doi: 10.5334/cpsy.164 (PMC13450271; doi:10.5334/cpsy.164)
Supplement: Peer review history

# Reviewer report: Round 1, Reviewer B

## Recommendation:

Accept with Major Revision

## Comments/Explanation (required). Please include references to any minor or major revisions required, as well as addressing any issues with language clarity. Authors will see these comments:

This paper investigates how decision-making behaviours (exploration/exploitation strategies) relate to brain microstructure. Participants completed a task in which they were asked to select across three possible bandits (trees) to obtain samples (apples) of varying value (apple size), to maximise their reward, in conditions of varying trial horizons (how many draws remained in a trial). Both behavioural metrics and computational model parameters indicated that long trial horizon favoured exploratory behaviours, where this strategy is beneficial to maximize reward, while short trial horizon favoured exploitative behaviours. Participants also completed a multi parametric mapping MRI protocol to derive MT, R1, and R2* maps. R1 values, indexing myelination, were associated with exploration parameters in trials where exploration is more beneficial (long horizon trials). MT values showed a negative association with the same metric in the right postcentral gyrus and superior parietal lobe in short horizon trials, meaning that higher myelination in these areas were associated with reduced exploration behaviour when this strategy was not beneficial. No significant associations were found with R2*.

This is a well written paper with a straightforward analytical approach. I have a few suggestions for the authors that may complement presented results and strengthen the conclusions drawn from the analyses.

1. The authors implement a computational model to quantify behaviour during the task. They also show that simpler metrics, extracted directly from subject performance (e.g., choice frequency) tend to go in the same direction as the computational model parameter used here. The authors could consider demonstrating potential advantages of their computational modelling strategy in detecting neurobiological effects. For example, are the reported significant clusters only detectable in association with the selected modelling parameter? Do brain-behaviour associations differ when using direct behavioural metrics?

2. The authors state in the introduction: “Examining cortical differences through microstructural imaging can potentially reveal the underlying neuroanatomical origins of these observed differences in behaviour and brain functional pathways.”, which suggests that the present study may combine all of these three elements (microstructure, brain function, behaviour). However, the link to functional pathways is missing here. Can the present findings be linked to networks involved in decision making in some way, perhaps if the subjects also underwent functional imaging or using meta-analytic maps from tools like NeuroSynth?

3. In the discussion, the authors state: “In contrast to voxel-based morphometry (VBM), which supplies indirect, relative differences in tissue densities and is sensitive to differences in processing choices, VBQ provides biophysically plausible parameters describing absolute microstructural variations in cortical tissues (Senjem et al. 2005).” Even if it provides biophysically plausible parameters, VBQ, just like any analytical approach, is also sensitive to processing choices. How did the authors ensure that their results were robust to such variations, including for instance smoothing and registration (surface-based instead of volumetric)?

4. As noted by the authors in their discussion “qMRI parameters vary across cortical depths”. This could be directly assessed in relation to task performance in the present work using surface-based methods rather than voxel-level analysis. Surface-based registration furthermore provides more precise spatial normalization in the neocortex, relating to my previous point on robustness of findings to pre-processing choices. I acknowledge this may extend beyond the scope of the present work, but this could be noted as a limitation, as the present work is blind to depth-specific effects given the processing pipeline it implements.

5. While the task design is described in sufficient detail, could the authors report what instructions were given to the participants to complete the task? I could not find this in the paper but perhaps I missed it.

6. The discussion could benefit from broader explorations of possible mechanisms for the link between myelination of uncovered regions and exploration/exploitation behaviour.

7. In the introduction, why do the authors describe cortical volume and thickness as qualitative metrics? They can both be quantified, perhaps the authors meant to say that their biological underpinnings are not established? This should be clarified.

# Reviewer report: Round 1, Reviewer F

## Recommendation:

Accept with Major Revision

## Comments/Explanation (required). Please include references to any minor or major revisions required, as well as addressing any issues with language clarity. Authors will see these comments:

In this work, Tyrer et al. use a multi-armed bandit task and multi-parameter mapping (MPM) to study relationships between brain myelin/iron content and exploration-exploitation decision making strategies. The authors first demonstrate that long horizon trials in the bandit task are associated with beneficial information seeking and increased computational model-derived measures of value-free and novelty-driven exploration. Next, using whole-brain multiple linear regression, the authors report significant relationships between greater value-free random exploration in long horizon trials and higher R1 in right PFC as well as between greater value-free random exploration in short horizon trials and lower MT saturation in somatosensory cortex. Overall, this is a methodologically rigorous set of analyses that will be of interest to the decision-making community and to scientists studying the functional consequences of cortical myelination.

The main strengths of this work include the use of a behavioral paradigm that modulates explore-exploit decisions via short/long trial horizons, a computational model that distinguishes between value-based, value-free, and novelty exploration, and the use of quantitative multi-parameter mapping to study cortical properties with high biological specificity. Main limitations include the small sample size for examining brain-cognition relationships, an underdeveloped conceptual model linking cortical myelination and iron to exploration, a degree of methodological ambiguity and the lack of publicly available code, and no external validation of computational model-derived exploration parameters.

Below I offer specific comments aimed at improving the clarity and robustness of the work:

Study rationale and conceptual model: I found the overall rationale for investigating relationships between quantitative MRI parameters and exploration-exploitation behaviors to be somewhat undeveloped. The authors describe how explore-exploit behaviors have been linked to noradrenergic and dopaminergic activity, functional pathways, and cortical thickness, and note that “inter-individual differences in cortical microstructures” may also be “driving this behavioural variability”. Yet there is no clear conceptual or neurobiological model put forth hypothesizing why the specific properties examined here with MPM—myelination and iron—may be important for explore-exploit decision making. I believe that part of the author’s rationale is that MPM measures are related to neurochemicals, as they write “the R2* quantitative contrast, which indexes the iron content of cortical tissues, has been repeatedly associated with noradrenergic and dopaminergic activity due to the accumulation of the neuromelanin-iron complex in noradrenergic neurons in the locus coeruleus, and dopaminergic neurons in the substantia nigra.” While it is true that R2*-indexed iron is related to neuromelanin in the LC and dopamine in the SN (and basal ganglia), R2* in the cortex is most strongly related to myeloarchitecture (e.g., Fukunaga et al., 2010, PNAS). Accordingly, how do cortical R2*, R1, and MT saturation provide insight into the “relationship between structural brain components and neurotransmitters with putative roles in decision-making” and what role may cortical myelin and iron play in exploration?

Quantitative MPM description and processing: I have a few questions regarding the MPM processing pipeline and interpretation of derived measures.

- The authors note that the hMRI toolbox was used to correct raw qMRI images for transmit (B1+) and receive (B1-) field inhomogeneity, yet they do not specify how. Was correction done using empirical data (e.g., the B1+ map and receive field sensitivity measurements) or a data-driven method (e.g., UNICORT)?

- The methods and results link MT saturation to “myeloarchitectural integrity” and R1 to “myelination”. Are these two distinct myelin-related constructs? How do myeloarchitectural integrity and myelination levels (and thus MT and R1) differ? This distinction is important for interpretating the results.

- The Discussion states that “To further investigate the contributions of subcortical microstructural variation to exploration behaviours, future studies may employ optimised qMRI sequences to specifically target subcortical regions of interest.” Can the authors expand on what these sequences may be and how they differentially target subcortical structures?

Behavioral findings: I found the clear change in participants’ behavior between short and long horizon trials to be interesting and compelling. It would be valuable to further test what forms of exploration account for most of the observed differences between short and long trials, and to understand when the transition from exploring to exploiting on long trials occurs. At present, the authors emphasize that “long horizon trials engendered robust value-free exploratory behaviors”. Did value-based random exploration increase between short and long trials? Did the authors compare the performance of their model to alternative computational models that parameterize exploration differently (e.g., directed and random, undirected only)? Were there individual differences in when participants switched from “exploring” on long trials (selecting lower-value but novel or informative bandits) to “exploiting”?

Statistical models: If I understand correctly, the authors included age, gender, BMI, TIV, and 15 behavioral measures (or 16 as in Table 2?) as predictors in multiple linear regressions with MPM measures as the outcome variables. Can the authors provide further context for why all parameters in Table 2 should be included in multiple regressions simultaneously? Do the main findings relating R1 and MT saturation to ε-greedy parameter estimates on long and short horizons, respectively, remain when ε-greedy is studied as a univariate behavioral predictor (while still controlling for nuisance covariates). Given that exploration has been associated with socioeconomic status (Decker et al., 2025, Nature Communications), do the main findings replicate when indices of socioeconomic position are included as nuisance covariates?

Computational measures of exploration: I appreciate the author’s use of a sophisticated computational model to quantitatively operationalize exploratory behavior. However, exploration parameters are not always generalizable/reliable across tasks and do not necessarily map onto non-task measurements of exploration tendencies (Eckstein et al., 2022, eLife; Witte et al., 2025, Scientific Reports). If the authors cannot show generalizability to an independent task or external validity, this consideration should be acknowledged in the Discussion.

# Reviewer report: Round 2, Reviewer A

## Recommendation:

Accept (No Revisions Necessary)

## Comments/Explanation (required). Please include references to any minor or major revisions required, as well as addressing any issues with language clarity. Authors will see these comments:

I thank the authors for their detailed response to my comments - all queries have been appropriately addressed.

# Reviewer report: Round 2, Reviewer B

## Recommendation:

Accept (No Revisions Necessary)

## Comments/Explanation (required). Please include references to any minor or major revisions required, as well as addressing any issues with language clarity. Authors will see these comments:

The authors have addressed all of my concerns. Congratulations on an interesting study! Signed - Valerie Sydnor

# Reviewer report: Round 1, Author Response

## Reply to Reviewer Comments

We sincerely thank the editor and reviewers for their thoughtful efforts and valuable feedback on our manuscript. The reviewers’ comments below are presented in black text, our responses are in blue, and quoted changes to the manuscript are displayed in quoted blue italics.

## Reviewer A:

This paper investigates how decision-making behaviours (exploration/exploitation strategies) relate to brain microstructure. Participants completed a task in which they were asked to select across three possible bandits (trees) to obtain samples (apples) of varying value (apple size), to maximise their reward, in conditions of varying trial horizons (how many draws remained in a trial). Both behavioural metrics and computational model parameters indicated that long trial horizon favoured exploratory behaviours, where this strategy is beneficial to maximize reward, while short trial horizon favoured exploitative behaviours. Participants also completed a multi parametric mapping MRI protocol to derive MT, R1, and R2* maps. R1 values, indexing myelination, were associated with exploration parameters in trials where exploration is more beneficial (long horizon trials). MT values showed a negative association with the same metric in the right postcentral gyrus and superior parietal lobe in short horizon trials, meaning that higher myelination in these areas were associated with reduced exploration behaviour when this strategy was not beneficial. No significant associations were found with R2*.

This is a well written paper with a straightforward analytical approach. I have a few suggestions for the authors that may complement presented results and strengthen the conclusions drawn from the analyses.

## Author’s response

Many thanks for your support of our work and for taking the time to review our paper. We believe that the points you raised in your review greatly increased the quality of our manuscript and we have done our best to improve our paper in response to the questions you have raised.

## Reviewer A:

1. The authors implement a computational model to quantify behaviour during the task. They also show that simpler metrics, extracted directly from subject performance (e.g., choice frequency) tend to go in the same direction as the computational model parameter used here. The authors could consider demonstrating potential advantages of their computational modelling strategy in detecting neurobiological effects. For example, are the reported significant clusters only detectable in association with the selected modelling parameter? Do brain-behaviour associations differ when using direct behavioural metrics?

## Author’s response

Thank you for this suggestion. We have now examined the correlations between simpler behavioural metrics, namely bandit choice frequency, and indices of cortical microstructures. We specifically looked at the frequency that each participant selected the low-value bandit, as this behavioural metric most strongly correlates (both statistically and behaviourally) with the ε-greedy parameter, i.e., value-free random exploration (please see Supplementary Figure 1 for partial correlation matrix displaying correlations between ε-greedy and low-value bandit selection frequencies in short and long horizon trials). In our original findings, we reported a significant positive correlation between R1 map values in the right superior frontal gyrus (rSFG) and ε-greedy parameter estimates specifically in long horizon trials (*k_E_* = 6539, *p* = 0.015, FWE corrected). However, when studying low-value bandit selection frequency in long horizon trials, we only observe a smaller positive correlation with R1 map values for a much smaller cluster in the region of the lateral ventricle (*k_E_* = 82, *p* = 0.030, uncorrected), and this correlation did not survive FWE correction.

In our original findings we also reported a significant negative correlation between ε-greedy parameter estimates specifically in short horizon trials and MT map values in the right postcentral gyrus (rPCG) (*k_E_* = 5840, *p* = 0.024, FWE corrected). When studying low-value bandit selection frequency in short horizon trials, we instead find negative correlations with MT map values with most significant clusters in the left putamen and left pallidum (left putamen: *k_E_* = 3949, *p* = 0.015 uncorrected; left pallidum: *k_E_* = 657, *p* = 0.018 uncorrected), however these associations also did not survive FWE correction. This therefore demonstrates that our computational model captures distinct neurobiological effects compared with those captured by simpler behavioural metrics, such as raw selection frequencies, and we have highlighted this in our Results section; please see added text below:

“*These significant clusters were only detectable in association with ε-greedy parameter estimates and were not detectable when examining bandit selection frequencies (see Supplementary Figure 4, Supplementary Tables 1-2), suggesting that our computational model captures distinct neurobiological effects compared with those captured by simpler behavioural metrics, such as raw selection frequencies*.”

To clearly demonstrate the advantages of our computational modelling strategy, we have added the following text to the manuscript:

“*Here, we employ a computational model to quantitatively describe specific psychological traits such as value-free random exploration, by combining the resource-demanding Thompson sampling with two computationally-light heuristic strategies. The utilisation of multiple different exploration behaviours, which all approximate an optimal exploration strategy, has been widely reported in human decision-making (Dubois et al., 2022; Gershman, 2018; Wilson et al., 2014). Examining simpler behavioural metrics alone, such as bandit selection frequencies, does not necessarily allow us to tease apart these different properties of exploration and may absorb shared variance from multiple forms of exploratory behaviour, for example directed versus random exploration*.”

We have also added a new Supplementary Figure (SF4) displaying the differences in significant clusters in R1 and MT maps between those correlated with ε-greedy and those correlated with simpler behavioural metrics, and have included a summary of the results from this control analysis in two supplementary tables (Supplementary Tables 1-2):

**SF4:** Comparison of main VBQ findings examining associations between cortical microstructures and ε-greedy parameter estimates (left), versus the simpler behavioural metric of bandit selection frequency (right).

**Supplementary Table 1:** Summary of whole brain VBQ results: positive correlation between R1 map values and low-value bandit selection frequency in long horizon trials. Clusters are ordered by FWE-corrected p value.


R1 Whole Brain Clusters for Raw Behavioural Data							

Region	*k*	p(FWE-corr)	p(uncorr.)	TFCE	x	y	z

Left lateral ventricle	1	1.00	0.049	132.53	–24.0	–52.8	13.6

Left lateral ventricle	1	1.00	0.049	132.04	–23.2	–51.2	12.8

Right lateral ventricle	19	1.00	0.043	123.99	34.4	–42.4	3.2

Right lateral ventricle	82	1.00	0.030	81.92	23.2	–30.4	24


**Supplementary Table 2:** Summary of top ten whole brain VBQ results: negative correlation between MT Saturation and low-value bandit selection frequency in short horizon trials. Clusters are ordered by FWE-corrected p value. DC = diencephalon; OrIFG = orbital part of inferior frontal gyrus.


MT Whole Brain Clusters for Raw Behavioural Data							
Region	*k*	p(FWE-corr)	p(uncorr.)	TFCE	x	y	z

Left putamen	3949	0.200	0.015	1207.01	–33.6	–6.4	2.4

Left pallidum	657	0.208	0.018	1177.95	–18.4	–10.4	–2.4

Right basal forebrain	3433	0.211	0.016	1167.07	20.8	2.4	–15.2

Right frontal operculum	1771	0.241	0.011	1073.67	40.8	16.8	10.4

Right posterior orbital gyrus	465	0.246	0.020	1057.30	24.0	16.0	–14.4

Left parahippocampal gyrus	3517	0.268	0.013	997.49	–12.8	–7.2	–29.6

Left ventral DC	549	0.295	0.019	932.45	–12.8	–20.0	–18.4

Left parahippocampal gyrus	1	0.313	0.019	890.73	–20.0	30.4	–28.8

Left parahippocampal gyrus	10	0.316	0.019	884.47	–24.0	–26.4	–29.6

Right OrIFG	1149	0.335	0.017	846.34	48.0	29.6	–10.4


## Reviewer A:

2. The authors state in the introduction: “Examining cortical differences through microstructural imaging can potentially reveal the underlying neuroanatomical origins of these observed differences in behaviour and brain functional pathways.”, which suggests that the present study may combine all of these three elements (microstructure, brain function, behaviour). However, the link to functional pathways is missing here. Can the present findings be linked to networks involved in decision making in some way, perhaps if the subjects also underwent functional imaging or using meta-analytic maps from tools like NeuroSynth?

## Author’s response

Thank you for this suggestion. We have now analysed meta-analytic coactivation maps using Neurosynth to elucidate how such cortical microstructural differences may link to functional pathways, and have added the following text to the Results section, in addition to a Supplementary figure and two Supplementary tables summarising our findings. We have also amended the above statement to refer to “*brain anatomical pathways*” rather than “*brain functional pathways*”.

“*Finally, we conducted a meta-analytic coactivation analysis of our findings in the right postcentral gyrus for MT map values and right SFG for R1 map values. To do this we utilised Neurosynth; a publicly-available meta-analytic database, using the right SFG and right postcentral gyrus peaks as seed regions. We generated meta-analytic maps displaying significant co-activation with our seed regions, in addition to associated functional and psychological terms from the literature. We sorted all associated terms with a non-zero reverse inference z-score by their posterior probability of appearing in a publication, given a pattern of activation (p(term | activation)). The top literature terms (max. ten) for each region are presented in Supplementary Tables 3-4 (see Supplementary Figure 6 for meta-analytic maps for each region)*.

*We found that the right postcentral gyrus displayed significant coactivation with the right primary visual cortex, and bilateral premotor regions. In terms of functional associations, the right postcentral gyrus was most commonly related to speech, in line with suggestions that the right postcentral gyrus is linked to sensory representations of the larynx (example top terms: “pitch”, “speech production”; “vocal”) (Belyk and Brown 2014). Previous work has also implicated functional activations in the right postcentral gyrus in implicit learning in autism spectrum disorder (Schipul and Just 2016). In contrast, the right SFG displayed significant coactivation with the right insula, in addition to left premotor/supplementary motor regions. The right SFG was primarily associated with motor-based meta-analytic terms (example top terms: “muscle”, “motor premotor”), and has been linked to error detection and inhibition of distractor interference (Chevrier and Schachar 2010; Xu et al. 2011)*.”

**SF6:** Meta-analytic analyses for right SFG and right PostCG seed regions identified through VBQ analyses, displaying areas of significant coactivation. SFG = superior frontal gyrus; PostCG = postcentral gyrus.

**Supplementary Table 3:** Top ten meta-analysis map associations for the right SFG, ordered by posterior probability.


Name	z-score	Posterior prob.	Func. conn. (r)	Meta-analytic coact. (r)

foot	8.35	0.95	0.48	0.19

muscle	6.02	0.92	0.23	0.13

suppressed	5.95	0.92	0.01	0

substantia	5.13	0.92	–0.05	–0.01

motor premotor	4.82	0.91	0.28	0.12

distractor	4.75	0.91	–0.01	–0.01

electrical	5.12	0.9	0.19	0.16

cortex dorsal	4.58	0.9	0.02	0.01

midbrain	4.9	0.88	–0.06	0

coordination	4.55	0.88	0.27	0.22


**Supplementary Table 4:** Meta-analysis map associations for the right postcentral gyrus, ordered by posterior probability.


Name	z-score	Posterior prob.	Func. conn. (r)	Meta-analytic coact. (r)

postcentral gyrus	6.54	0.89	0.14	0.2

pitch	5.57	0.89	0.19	0.26

speech production	5.4	0.89	0.35	0.44

taste	5.04	0.89	0.02	0.09

vocal	4.78	0.89	0.24	0.31

postcentral	5.49	0.85	0.1	0.26

multisensory	4.38	0.85	0.07	0.13


## Reviewer A:

3. In the discussion, the authors state: “In contrast to voxel-based morphometry (VBM), which supplies indirect, relative differences in tissue densities and is sensitive to differences in processing choices, VBQ provides biophysically plausible parameters describing absolute microstructural variations in cortical tissues (Senjem et al. 2005).” Even if it provides biophysically plausible parameters, VBQ, just like any analytical approach, is also sensitive to processing choices. How did the authors ensure that their results were robust to such variations, including for instance smoothing and registration (surface-based instead of volumetric)?

## Author’s response

Thank you for highlighting this important point. Indeed, like all analytical approaches, VBQ is sensitive to processing choices. Our original statement was intended to highlight the benefits of VBQ as opposed to VBM in generating biophysically-interpretable parameters rather than to imply that it is processing-invariant. Importantly, however, the VBQ pipeline employed here incorporates several features specifically designed to minimise sensitivity to such variations. For example, as detailed in the Methods of our original manuscript, our use of a unified segmentation-normalisation framework that has been specially adapted for VBQ (Ashburner and Friston 2005) ensures that registration is performed simultaneously with tissue classification, reducing the compounding of errors across processing steps. We also applied tissue-weighted smoothing utilising an optimised voxel-based quantification smoothing pipeline (see Draganski et al. 2011 for details). In contrast to voxel-based morphometry analysis, this VBQ smoothing approach aims to minimise partial volume effects and optimally preserves the quantitative values of the original qMRI images by not modulating the parameter maps to account for volume changes.

Regarding surface-based registration as an alternative approach: while we agree this is a valuable methodological direction, implementing and validating a surface-based VBQ pipeline constitutes a substantial methodological undertaking that falls outside the scope of the present study. We believe that the volumetric approach used in this study better addresses our specific research questions pertaining to whole-brain microstructural properties.

We do however acknowledge that the susceptibility of VBQ to processing choices presents a potential limitation to the current study, and have added the following text to our Discussion:

“*As with any voxel-based methodology, our findings may be subject to some degree of sensitivity to processing choices, including spatial normalisation and smoothing parameters. To mitigate this, we employed a unified segmentation-normalisation framework in which registration and tissue classification are performed simultaneously (Ashburner and Friston 2005), and utilised a smoothing approach specifically optimised for VBQ to minimise partial volume effects and optimally preserve the quantitative values of the original qMRI images (Draganski et al. 2011)*.”

## Reviewer A:

4. As noted by the authors in their discussion “qMRI parameters vary across cortical depths”. This could be directly assessed in relation to task performance in the present work using surface-based methods rather than voxel-level analysis. Surface-based registration furthermore provides more precise spatial normalization in the neocortex, relating to my previous point on robustness of findings to pre-processing choices. I acknowledge this may extend beyond the scope of the present work, but this could be noted as a limitation, as the present work is blind to depth-specific effects given the processing pipeline it implements.

## Author’s response

Thank you for highlighting this important point. This is indeed beyond the scope of the current work; we have, however, added this as a limitation in the Discussion:

“*The variation in qMRl parameters across cortical depths presents an additional limitation, as the processing pipeline employed here does not account for depth-specific effects. Future work could build upon our voxel-level analyses by utilising surface-based methods, such as surface-based registration, to examine such depth-specific effects and also improve the precision of spatial normalisation (Khan et al. 2011)*.”

## Reviewer A:

5. While the task design is described in sufficient detail, could the authors report what instructions were given to the participants to complete the task? I could not find this in the paper but perhaps I missed it.

## Author’s response

Before the main task started, participants were shown instructions for the task alongside visual examples of bandits (trees bearing apples) and rewards (apples). Participants were instructed to collect the biggest apples before the end of each trial (sunset). We have added this clarification to the Methods:

“*Participants were instructed to collect the biggest apples before the end of each trial (sunset)*.”

The detailed instructions presented as part of the task tutorial are as follows, and this has been added to the Supplementary Information:

“*Apples come in different shades and sizes. You need to help us collect the BIGGEST ones before sunset. You can only pick apples until sunset. Sometimes you will start at noon and will pick six apples, sometimes you will start in the afternoon and will pick only one apple. On each day you will collect apples from new trees. Some of the trees are better than others. To help you, some apples were already picked up before you arrived. These are the three different types of trees. Each tree produces an apple with a corresponding colour, but it’s their size that matters! For example, if we look at the apples produced, the yellow tree seems to be better on this day because it produces bigger apples ON AVERAGE. This is a big apple from the yellow tree. This is a medium apple from the red tree. This is a small apple from the yellow tree. This is a small apple from the blue tree. And finally this is a big apple from the blue tree. You will press the key 1, 2, or 3 to select the tree you wish to pick an apple from. If the sun is here, it is still early and you will be able to pick six apples. If the sun is almost set, you will only be able to pick one apple. At the end of each day, you will see the amount of juice extracted from the apples you collected. The juice will be poured in a small glass if one apple was picked, and in a big glass if six apples were picked*.”

## Reviewer A:

6. The discussion could benefit from broader explorations of possible mechanisms for the link between myelination of uncovered regions and exploration/exploitation behaviour.

## Author’s response

We have now added the following paragraph discussing possible mechanisms for the association between exploration-exploitation behaviours and myelination of uncovered regions to the Discussion:

“*The association between variations in cortical microstructures and exploration-exploitation behaviours may stem from various mechanisms. Myelination, for instance, is responsible for increased conduction velocity of axonal electrical signals, and is therefore crucial for facilitating fast and efficient neural signalling (Nave 2010; Purves et al. 2001). Speculatively, this improved signalling efficiency may enable more flexibility in individuals’ internal models of the environment, therefore facilitating switching to exploratory behavioural strategies in response to environmental instability. The right SFG has been previously implicated with exploratory decision-making (Laureiro-Martínez et al. 2014; Wyatt et al. 2024), whereas the right postcentral gyrus has been linked to sensory gating and regulation of task-relevant sensorimotor responses (Meehan & Staines 2007; Staines et al. 2002). Within this framework, increased myelination in the right SFG may indicate enhanced efficiency in the neural circuits supporting exploratory policy implementation, consistent with higher ε-greedy parameter estimates in trials where exploration is beneficial. In contrast, greater indices of myeloarchitecture in the right postcentral gyrus may improve the suppression of task-irrelevant sensorimotor signals and the regulation of stochastic response tendencies, thereby reducing unnecessary random exploration and supporting more stable exploitative behaviour in trials where exploration is not beneficial*.”

## Reviewer A:

7. In the introduction, why do the authors describe cortical volume and thickness as qualitative metrics? They can both be quantified, perhaps the authors meant to say that their biological underpinnings are not established? This should be clarified.

## Author’s response

You are correct that this statement was poorly worded; cortical volume and thickness are indeed quantifiable. Our intention was to distinguish conventional morphometric measures which may stem from various microstructural processes, from quantitative MRI parameters that are expressed in calibrated, non-arbitrary units and have more direct biophysical interpretations. We have revised the sentence in the Introduction to clarify this distinction:

“*These indices extend beyond conventional morphometric measures such as cortical volume or thickness to index variation in quantitative, non-arbitrary units with direct histological correlates*.”

## Reviewer F:

In this work, Tyrer et al. use a multi-armed bandit task and multi-parameter mapping (MPM) to study relationships between brain myelin/iron content and exploration-exploitation decision making strategies. The authors first demonstrate that long horizon trials in the bandit task are associated with beneficial information seeking and increased computational model-derived measures of value-free and novelty-driven exploration. Next, using whole-brain multiple linear regression, the authors report significant relationships between greater value-free random exploration in long horizon trials and higher R1 in right PFC as well as between greater value-free random exploration in short horizon trials and lower MT saturation in somatosensory cortex. Overall, this is a methodologically rigorous set of analyses that will be of interest to the decision-making community and to scientists studying the functional consequences of cortical myelination.

## Author’s response

Thank you for your appreciation of our work and for taking the time to review our paper. We greatly appreciate your comments and believe that your points have significantly improved the quality of the work.

## Reviewer F:

The main strengths of this work include the use of a behavioral paradigm that modulates explore-exploit decisions via short/long trial horizons, a computational model that distinguishes between value-based, value-free, and novelty exploration, and the use of quantitative multi-parameter mapping to study cortical properties with high biological specificity. Main limitations include the small sample size for examining brain-cognition relationships, an underdeveloped conceptual model linking cortical myelination and iron to exploration, a degree of methodological ambiguity and the lack of publicly available code, and no external validation of computational model-derived exploration parameters.

Below I offer specific comments aimed at improving the clarity and robustness of the work:

Study rationale and conceptual model: I found the overall rationale for investigating relationships between quantitative MRI parameters and exploration-exploitation behaviors to be somewhat undeveloped. The authors describe how explore-exploit behaviors have been linked to noradrenergic and dopaminergic activity, functional pathways, and cortical thickness, and note that “inter-individual differences in cortical microstructures” may also be “driving this behavioural variability”. Yet there is no clear conceptual or neurobiological model put forth hypothesizing why the specific properties examined here with MPM—myelination and iron—may be important for explore-exploit decision making. I believe that part of the author’s rationale is that MPM measures are related to neurochemicals, as they write “the R2* quantitative contrast, which indexes the iron content of cortical tissues, has been repeatedly associated with noradrenergic and dopaminergic activity due to the accumulation of the neuromelanin-iron complex in noradrenergic neurons in the locus coeruleus, and dopaminergic neurons in the substantia nigra.” While it is true that R2*-indexed iron is related to neuromelanin in the LC and dopamine in the SN (and basal ganglia), R2* in the cortex is most strongly related to myeloarchitecture (e.g., Fukunaga et al., 2010, PNAS). Accordingly, how do cortical R2*, R1, and MT saturation provide insight into the “relationship between structural brain components and neurotransmitters with putative roles in decision-making” and what role may cortical myelin and iron play in exploration?

## Author’s response

Thank you for highlighting this. We have now added the following three statements throughout the Introduction to address this:

“*Cortical microstructures such as myeloarchitecture play a vital role in the efficiency of neural signalling, by improving conduction velocity and reducing signal-to-noise ratio (Nave, 2010; Purves et al., 2001)*.”

“*However, these neuromodulatory influences are expressed through distributed cortical circuits, suggesting that variability in the structural properties of such circuits controls how neurochemical signals are integrated and translated into behaviour*.”

“*More specifically, exploration-exploitation behaviours rely on the balance between flexible, stochastic policy control, often associated with prefrontal networks, and more stable, value-based action selection frequently attributed to parietal and sensorimotor regions (Daw et al., 2006; Klein-Flügge & Bestmann, 2012). Such processes are likely to rely heavily on the efficiency of neural signalling and underlying structural connectivity*.”

To address another related concern of Reviewer 1, we have also contextualised our findings in relation to this conceptual model in the Discussion:

“*The association between variations in cortical microstructures and exploration-exploitation behaviours may stem from various mechanisms. Myelination, for instance, is responsible for increased conduction velocity of axonal electrical signals, and is therefore crucial for facilitating fast and efficient neural signalling (Nave, 2010; Purves et al., 2001). Speculatively, this improved signalling efficiency may enable more flexibility in individuals’ internal models of the environment, therefore facilitating switching to exploratory behavioural strategies in response to environmental instability. The right SFG has been previously implicated with exploratory decision-making (Laureiro-Martínez et al., 2014; Wyatt et al., 2024), whereas the right postcentral gyrus has been linked to sensory gating and regulation of task-relevant sensorimotor responses (Meehan & Staines, 2007; Staines et al., 2002). Within this framework, increased myelination in the right SFG may indicate enhanced efficiency in the neural circuits supporting exploratory policy implementation, consistent with higher ε-greedy parameter estimates in trials where exploration is beneficial. In contrast, greater indices of myeloarchitecture in the right postcentral gyrus may improve the suppression of task-irrelevant sensorimotor signals and the regulation of stochastic response tendencies, thereby reducing unnecessary random exploration and supporting more stable exploitative behaviour in trials where exploration is not beneficial*.”

## Reviewer F:

Quantitative MPM description and processing: I have a few questions regarding the MPM processing pipeline and interpretation of derived measures.

- The authors note that the hMRI toolbox was used to correct raw qMRI images for transmit (B1+) and receive (B1-) field inhomogeneity, yet they do not specify how. Was correction done using empirical data (e.g., the B1+ map and receive field sensitivity measurements) or a data-driven method (e.g., UNICORT)?

## Author’s response

The correction was executed using empirical data, i.e., the RF sensitivity map and B1 maps collected from each participant. We have now clarified this detail in the Methods:

“*This correction was executed using empirical data, i.e., the RF sensitivity map and B1 maps collected from each participant*.”

## Reviewer F:

- The methods and results link MT saturation to “myeloarchitectural integrity” and R1 to “myelination”. Are these two distinct myelin-related constructs? How do myeloarchitectural integrity and myelination levels (and thus MT and R1) differ? This distinction is important for interpretating the results.

## Author’s response

Thank you for highlighting this. We have now included a clarification in the Methods section to better distinguish MT saturation and R1 map types:

“*MT saturation and R1 both reflect qualities of myelin, however they index different underlying biophysical properties (Filo et al., 2019; Weiskopf et al., 2021). R1 is predominantly shaped by tissue composition, water content, and iron, whereas MT saturation indexes macromolecular content through magnetisation transfer. As a result, subtle variations in microstructural myelin components may exert a greater effect on one map type compared with the other. However, the in-depth understanding of the neuroarchitecture-to-MPM contrast association remains an active research area, and some biophysical constructs contribute to both map types*.”

## Reviewer F:

- The Discussion states that “To further investigate the contributions of subcortical microstructural variation to exploration behaviours, future studies may employ optimised qMRI sequences to specifically target subcortical regions of interest.” Can the authors expand on what these sequences may be and how they differentially target subcortical structures?

## Author’s response

We have now specified in more detail some examples of sequences optimised for imaging subcortical regions of interest:

“*For example, multiple-measurement protocols have been previously employed to improve the low signal-to-noise ratio typically linked to the high spatial resolution necessary to image smaller subcortical structures, such as the locus coeruleus and substantia nigra (Chen et al. 2014)*.”

## Reviewer F:

Behavioral findings: I found the clear change in participants’ behavior between short and long horizon trials to be interesting and compelling. It would be valuable to further test what forms of exploration account for most of the observed differences between short and long trials, and to understand when the transition from exploring to exploiting on long trials occurs. At present, the authors emphasize that “long horizon trials engendered robust value-free exploratory behaviors”. Did value-based random exploration increase between short and long trials?

## Author’s response

The model employed in this study (Thompson model with value-free exploration (ε-greedy) and novelty exploration (novelty bonus η)), captures uncertainty-driven value-based random exploration in the prior variance (σ0) parameter. As shown in Figure 2C, the prior variance for long horizon trials was significantly greater than that for short horizon trials, which supports our conclusions that the long horizon promotes the exploration strategies examined in this study. We have now clarified in the Results section of our manuscript the meaning of the prior variance (a0) parameter, see below (text underlined is new added text):

“*Additionally, participants exhibited higher prior variances, i.e., **uncertainty-driven value-based random exploration**, or greater uncertainty about a bandit’s mean before seeing any samples…*”

## Reviewer F:

Did the authors compare the performance of their model to alternative computational models that parameterize exploration differently (e.g., directed and random, undirected only)? Were there individual differences in when participants switched from “exploring” on long trials (selecting lower-value but novel or informative bandits) to “exploiting”?

## Author’s response

Previous validation work examining the same task in a different sample (Dubois et al. 2021, *eLife*) compared multiple computational models, consisting of three base models: the Upper Confidence Bound (UCB) model which includes the UCB algorithm and a softmax choice function; the Thompson model which employs Thompson sampling; and a hybrid model which combined the UCB model and the Thompson model. These computationally-demanding models were compared both with and without the addition of simpler heuristic exploration strategies, i.e., value-free random exploration (ε-greedy), and novelty exploration (novelty bonus η).

Model comparison was performed using the Bayesian Information Criterion (BIC). Model fitting was performed using the maximum a posteriori probability (MAP) estimate, which allows incorporation of prior beliefs. All the parameters besides participants’ initial estimate of a bandit’s mean (Q0; prior mean) and the contribution of each model in the hybrid model (w) were free to vary as a function of the horizon as they capture different exploration forms. The Thompson model with both the ε-greedy parameter and the novelty bonus n proved to be the winning model, which was employed in the current study. Please see below Figure 4 from our previous work, Dubois et al. (2021), which compared models incorporating UCB, Thompson sampling, an ε-greedy algorithm and the novelty bonus:

**Figure 4:** **Dubois et al. (2021): Subjects use a mixture of exploration strategies.** (**a**) A 10-fold cross-validation of the likelihood of held-out data was used for model selection (chance level = 33.3%). The Thompson model with both the ε-greedy parameter and the novelty bonus n best predicted held-out data (b) Model simulation with 4^7^ simulations predicted good recoverability of model parameters; σ0 is the prior variance and Q0 is the prior mean. 1 stands for short horizon- and 2 for long horizon-specific parameters.

We have now elaborated upon our justification for employing the Thompson + ε + η model in the Methods section, please see added text below:

“*This previous validation work examining the same task in a different sample (Dubois et al. 2021) compared multiple computational models, consisting of three base models: the Upper Confidence Bound (UCB) model which includes the UCB algorithm and a softmax choice function; the Thompson model which employs Thompson sampling; and a hybrid model which combined the UCB model and the Thompson model. These computationally-demanding models were compared both with and without the addition of simpler heuristic exploration strategies, i.e., value-free random exploration (**ε**-greedy), and novelty exploration (novelty bonus η). Here, we employ the winning model from this model comparison, i.e., the model with the greatest held-out data likelihood (%): the Thompson model with the addition of ε-greedy and novelty bonus η*.”

Regarding the second point relating to individual differences in when participants switched from exploring to exploiting in long horizon trials: unfortunately we are unable to conduct this analysis as the data required (i.e., trial-wise bandit selections for all time steps within trials) were not retained as part of the original study design. We acknowledge that this analysis would provide valuable additional insight, and we have noted as such in the revised manuscript, please see text added to the Discussion below:

“*Additionally, we focused primarily on which bandit participants selected in their initial choice within a trial. Future work may also investigate at which point within long horizon trials participants switched from exploratory (i.e., selecting lower-value but novel or informative bandits) to exploitatory strategies to probe how cortical microstructures may drive individual differences in strategy switching*.”

## Reviewer F:

Statistical models: If I understand correctly, the authors included age, gender, BMI, TIV, and 15 behavioral measures (or 16 as in Table 2?) as predictors in multiple linear regressions with MPM measures as the outcome variables. Can the authors provide further context for why all parameters in Table 2 should be included in multiple regressions simultaneously? Do the main findings relating R1 and MT saturation to ε-greedy parameter estimates on long and short horizons, respectively, remain when ε-greedy is studied as a univariate behavioral predictor (while still controlling for nuisance covariates).

## Author’s response

Apologies; there are indeed 16 predictors in our multiple linear regressions. The ‘15’ in the Methods was a mistype and has now been corrected.

Including all 16 model parameters in our multiple regressions accounts for the behavioural and statistical correlation between ε-greedy and the remaining model parameter estimates. As shown in Supplementary Figure 1, ε-greedy exhibits low-to-moderate correlations with other parameters included in our design matrix. Therefore, studying ε-greedy as a univariate predictor may result in the absorption of shared variance from correlated predictors, such as prior variances.

When studying ε-greedy as a univariate predictor while still controlling for nuisance covariates, we still observe a significant positive correlation between ε-greedy parameter estimates in long horizon trials and R1 map values in the right superior frontal gyrus (rSFG), but at a lower significance level (p = 0.011, uncorrected). The rSFG also remained the largest (*k_E_* = 1525) and most significantly correlated cluster in this contrast when studying ε-greedy as a univariate predictor. For MT map values in the right postcentral gyrus (rPCG), we also still observe a significant negative correlation with ε-greedy parameter estimates in short horizon trials, also at a lower significance level (p = 0.010, uncorrected). The rPCG was the second largest cluster in this contrast (*k_E_* = 96), with the largest cluster located in the right visual cortex. This demonstrates that while these correlations are less significant when studying ε-greedy as a univariate predictor, the underlying relationship between ε-greedy and R1 and MT map values remain. When examining ε-greedy as a univariate predictor, the variance explained is naturally reduced compared to studying ε-greedy while controlling for additional model parameters, as shared variance from correlated model parameters, for example prior variances which capture different forms of exploratory behaviour, may be absorbed by the ε-greedy predictor.

By including all 16 model parameter estimates simultaneously, we were better able to isolate the unique variance associated with value-free random exploration specifically (indexed by ε-greedy) rather than conflating it with correlated model parameters.

We now present the differences between the full model (including all 16 regressors plus nuisance covariates) and reduced model (including ε-greedy as a univariate predictor plus nuisance covariates) in an additional Supplementary Figure (SF5), please see below:

**SF5:** Comparison of main VBQ findings for the original model comprising 16 regressors of interest and four nuisance covariates (age, gender, BMI, and TIV), and a reduced model comprising ε-greedy for short and long horizon trials only, plus the same four nuisance covariates.

## Reviewer F:

Given that exploration has been associated with socioeconomic status (Decker et al., 2025, Nature Communications), do the main findings replicate when indices of socioeconomic position are included as nuisance covariates?

## Author’s response

We did not directly collect demographic data pertaining to participants’ socioeconomic status, however all participants were predominantly young people (18-30) recruited from the Aarhus University student population in Denmark, which naturally consists of reduced socioeconomic variability compared with a community sample. We have now added the following statement in the Discussion highlighting this as a limitation:

“*Demographic factors such as socioeconomic status have been previously associated with exploration (Decker et al. 2025) and may therefore play an important role in individual differences in exploration-exploitation behaviours. The absence of such demographic data presents a potential limitation for this study, however our sample was primarily young adults recruited from the local University student population which in itself has relatively low socioeconomic variability compared to a community sample. Future work may employ exploratory factor analyses linking exploration behaviours and a range of lifestyle and demographic factors, including socioeconomic status*.”

## Reviewer F:

Computational measures of exploration: I appreciate the author’s use of a sophisticated computational model to quantitatively operationalize exploratory behavior. However, exploration parameters are not always generalizable/reliable across tasks and do not necessarily map onto non-task measurements of exploration tendencies (Eckstein et al., 2022, eLife; Witte et al., 2025, Scientific Reports). If the authors cannot show generalizability to an independent task or external validity, this consideration should be acknowledged in the Discussion.

## Author’s response

Thank you for highlighting this point. The exploration parameters in our model do not denote general exploration, rather they index specific exploratory behaviours; ε-greedy for example specifically denotes value-free random exploration, and the novelty bonus n indexes novelty exploration. As shown in the partial correlation matrix in Supplementary Figure 1, there is no correlation between ε-greedy and novelty bonus η, supporting our assertion that these parameters capture distinct exploratory processes.

However, our previous work employing the same computational model in the same multi-armed bandit task also collected self-report survey scores in the Barratt Impulsiveness Scale (BIS), and Adult ADHD Self-Report Scale (ASRS). We demonstrated significant positive correlations between ε-greedy parameter estimates in long horizon trials and general impulsivity, motor impulsivity (subscale of BIS), ADHD, and ADHD hyperactivity-impulsivity (subscale of ASRS) scores (Dubois and Hauser, 2022). Based on previous theoretical and empirical literature, exploration has been associated with increased impulsivity, particularly in ADHD (Hauser et al. 2014; Williams & Taylor 2006). We built upon this work by demonstrating that value-free random exploration specifically is increased in ADHD, while associations were not found for other forms of exploration. This finding is congruent with the idea of impulsivity as ‘acting on a whim’, as value-free random exploration disregards all existing information and is therefore the least computationally-demanding.

We do however acknowledge that the lack of generalisability of these exploration parameters to other tasks presents a limitation, and we have therefore added the following text to the Discussion:

“*Previous research has indicated that exploration model parameters may lack generalisability (Eckstein et al. 2022; Witte et al. 2025), which presents a possible limitation to the current study. However, the exploration parameters in our model do not denote general exploration, rather they index specific exploratory behaviours; **ε**-greedy for example specifically denotes value-free random exploration, and the novelty bonus n indexes novelty exploration. Our previous study (Dubois and Hauser 2022) employing the same computational model and task in a different sample also collected self-report survey scores in the Barratt Impulsiveness Scale (BIS), and Adult ADHD Self-Report Scale (ASRS). This work demonstrated significant positive correlations between **ε**-greedy parameter estimates in long horizon trials and general impulsivity, motor impulsivity (subscale of BIS), ADHD, and ADHD hyperactivity-impulsivity (subscale of ASRS) scores (Dubois and Hauser, 2022). This builds upon previous findings linking exploration to increased impulsivity, particularly in ADHD (Hauser et al. 2014; Williams & Taylor 2006), by demonstrating that value-free random exploration specifically is increased in ADHD, while associations were not found for other forms of exploration. This finding is congruent with the idea of impulsivity as ‘acting on a whim’, as value-free random exploration disregards all existing information and is therefore the least computationally-demanding. Future work may examine the association between variations in cortical microstructures and self-report measures of impulsivity, and how these metrics interact with various exploratory strategies*.”
